# The Role of Mast Cells in Healing Purulent Wounds Using a Drug from the Polyhexamethylene Guanidine Group with the Antiseptic Polyhexanide: An Ultrastructural Study

**DOI:** 10.3390/ijms262110405

**Published:** 2025-10-26

**Authors:** Irina Chekmareva, Atim Emaimo John, Andrey Kostin, Alexander Alekhnovich, Artem Volodkin, Ilya Klabukov, Denis Baranovskii, Viktoria Shishkina, Igor Buchwalow, Markus Tiemann, Dmitrii Atiakshin

**Affiliations:** 1 Research and Educational Resource Center for Immunophenotyping, Digital Spatial Profiling and Ultrastructural Analysis Innovative Technologies, RUDN University, 117198 Moscow, Russia; chia236@mail.ru (I.C.); emaimo_dzhon_a@pfur.ru (A.E.J.); andocrey@mail.ru (A.K.); alekhnovich_av@pfur.ru (A.A.); volodkin-av@rudn.ru (A.V.); buchwalow@pathologie-hh.de (I.B.); 2 Federal State Budgetary Institution “National Medical Research Center of Surgery Named after A. Vishnevsky”, Ministry of Health of the Russian Federation, 115093 Moscow, Russia; 3 Department of Regenerative Medicine, National Medical Research Radiological Centre of the Ministry of Health of the Russian Federation, Koroleva st, 249036 Obninsk, Russiadoc.baranovsky@gmail.com (D.B.); 4 Research Institute of Experimental Biology and Medicine, Burdenko Voronezh State Medical University, 394036 Voronezh, Russia; 4128069@gmail.com; 5 Institute for Hematopathology, Fangdieckstr. 75a, 22547 Hamburg, Germany; mtiemann@hp-hamburg.de

**Keywords:** purulent wound, mast cells, inflammation, re-epithelization healing, secretory pathways, ultrastructural analysis

## Abstract

Wound healing is a delicately regulated pathophysiological process based on molecular, cellular, and tissue interactions. Mast cells (MCs) are involved in the reparative process in all phases of wound healing, which indicates their general significance in reparative processes. The structural and functional changes in the MCs during the healing process correspond to the phase of the wound process and determine its course. In the inflammatory phase, rapid whole-granular degranulation of MCs with the secretion of biologically active proinflammatory substances that have a stimulating effect on inflammatory cells prevailed. In the proliferation phase, the maximum number of MCs per unit area of wound tissue and the maximum degranulation index were noted. In the phase of granulated tissue remodeling, the amount and functional activity of MCs sharply decrease, which contributes to the completion of the healing process with the formation of a fully fledged normotrophic scar. The gradual degranulation of MCs was characteristic of the proliferation and remodeling phases. The treatment of purulent wounds with a drug from the polyhexamethylene guanidine group with the antiseptic polyhexanide 0.1% contributed to a temporary shift in the phases of the wound process while maintaining its general patterns, while the activation of the process occurred at an earlier time than in the control group of animals without local treatment. The results obtained showed that the use of a drug from the polyhexamethylene guanidine group with the antiseptic polyhexanide 0.1% for the treatment of purulent wounds quickly stops the inflammatory response and creates conditions for the development of the reparative abilities of granulation tissue cells, and primarily, mast cells.

## 1. Introduction

Wound healing is a finely regulated pathophysiological process based on molecular, cellular, and tissue interactions, which includes interactions between resident cells, cells migrating to the area of injury, and intercellular matrix molecules—cytokines and chemokines [1]. The process includes four distinct and overlapping phases, hemostasis, inflammation, proliferation, and remodeling, which encompass a number of complex mechanisms, including the regulation of inflammation, angiogenesis, extracellular matrix remodeling, and cell proliferation, differentiation, and migration [2].

The morphological aspects of various wound cellular components involvement in the recovery process are extremely important, since different degrees of cell involvement in the reparative process entail a change in their structural and functional state, which ultimately reflects on the healing time of wounds and the quality of regenerate [3]. Mast cells are involved in the reparative process in all phases of wound healing, which indicates their significance in reparative processes [4,5,6,7,8]. The key mediators that are formed in MCs are serine proteases, tryptase, chymase, cathepsin G, histamine, heparin, serotonin, acid hydrolases, tumor necrosis factor-α (TNF-α), and interleukin-16 [9,10,11,12]. MCs are activated by cytokines and secrete proinflammatory mediators [13,14,15,16,17,18]. Heparin, for example, is a universal regulator of various processes including antithrombotic, anticoagulant, fibrinolytic, antimicrobial, antiviral, immunomodulatory, anti-inflammatory, antitoxic, antihistamine, antiallergic (inhibits the development of tumor necrosis factor and interleukin-4, and suppresses leukocyte infiltration of tissues) [19,20,21,22,23]. Tryptase, derived from MCs is a potent chemoattractant for neutrophils. It stimulates epithelial cell proliferation and IL-8 secretion, and increases epithelial expression of the intercellular adhesion molecule-1 [24,25,26].

Histamine plays a role in a variety of physiologic and pathologic processes such as cell proliferation, differentiation, hematopoiesis, vascular permeability, embryogenesis, tissue regeneration, and wound healing [9]. MCs are involved in first-line protection against pathogens and respond to various exogenous signals from bacteria through recognition receptors such as Toll-like receptors and immunoglobulins [27,28,29].

Once activated, mast cells promote angiogenesis in wounds by producing pro-angiogenic mediators such as heparin, histamine, major fibroblast growth factor (bFGF), vascular endothelial growth factor (VEGF), and various cytokines, such as tumor necrosis factor-α (TNF-α) and interleukin-8 (IL-8). VEGF, TGF-β, tryptase, and chymase stimulate angiogenesis, while heparin inhibits it [4,30,31,32].

MCs promotes collagen synthesis by activating fibroblasts. Growth factors secreted by MCs, such as TGF-β1 and FGF, regulate collagen synthesis. Tryptase causes the active movement of fibroblastic differon cells, mitotic division, and the stimulation of collagen protein synthesis, which leads to the proliferation of connective tissue [33,34,35]. Histamine, on the other hand, produces proteolytic enzymes and is involved in the remodeling of the extracellular matrix [6,9,36]. Thus, by releasing the contents of granules, MCs can affect neighboring cells and form a local microenvironment depending on the phase of the wound process. Harmonization and timely change in MC activity are the key to the uncomplicated flow of the wound process and normotrophic scar formation during wound healing by secondary tension.

During the healing of purulent wounds, there are shifts in the phases of wound processes. The phases are layered, and changes are observed in the intercellular and cell–matrix interactions, which undoubtedly affects the structural and functional state of the wound cells, and, in particular, MCs.

The use of modern highly effective antiseptics in the local treatment of purulent wounds contributes to the effective cleaning of wounds from biofilms, which can form in an infected wound within 2–4 days, depending on the types of bacteria and growth conditions. Biofilms maintain the inflammatory process and inhibit reparative processes in the wound. The polyhexamethylene-guanidine-group-based medication with the antiseptic polyhexanide 0.1% compromises the wholeness of the biofilm, acts on bacterial cell membranes, increases their permeability on account of the surface-active component in the cleaning composition, not only prevents the formation of microbial biofilms, but also dissolves the organic matrix of the biofilm [37,38].

However, no nanostructural analysis of the involvement of mast cells in wound healing has been performed using new generation antiseptics, including polyhexanide solution until now. Therefore, in this study, a focus was made on the structural and functional state of MCs in the treatment of purulent wounds, experimenting with a drug from the polyhexamethylene guanidine group in combination with the antiseptic polyhexanide 0.1%, including electron microscopic features of intercellular and cell–matrix interactions.

## 2. Results

In the second group, where purulent wounds were treated with a drug from the polyhexamethylene guanidine in combination with the antiseptic polyhexanide 0.1%, the level of microbial contamination significantly decreased on day 7, and by day 12, the bacteriological cultures were negative in all the experimental subjects. Whereas, in the control group, a high degree of contamination (more than 10^5^ CFU/g) of purulent wounds was observed throughout the observation period. The level of microbial contamination of wounds remained higher than critical on day 12 for 3 out of 5 subjects (see Table 1).

The filling of wound defects with mature granulated tissue occurred earlier in the experimental group than in the control group of animals (*p* < 0.05) (see Table 2). The average area of defects in group 1 (control) decreased from 421.6 ± 0.9 mm^2^ to 2.50850 ± 4.2 mm^2^ in twelve days, in group 2 (experimental)—from 421.2 ± 0.9 to 150.8 ± 3.6 mm^2^ (*p* < 0.05) (see Table 3). The average healing rate in group 2 was 5.8 ± 0.2% of the area in one day, while in group 1, it was 4.0 ± 0.11% (*p* < 0.05).

Thus, in group 2, local treatment of wound with a drug from the group of polyhexamethylene guanidine in combination with the antiseptic polyhexanide 0.1% showed effective cleaning of wound defects from microbial and cellular detritis, activation of the growth of granulation tissue, the start of the regional epithelialization process, and wound defect contraction, which led to wound healing earlier, compared to the control group.

### Morphological Study

**Figure 1:**
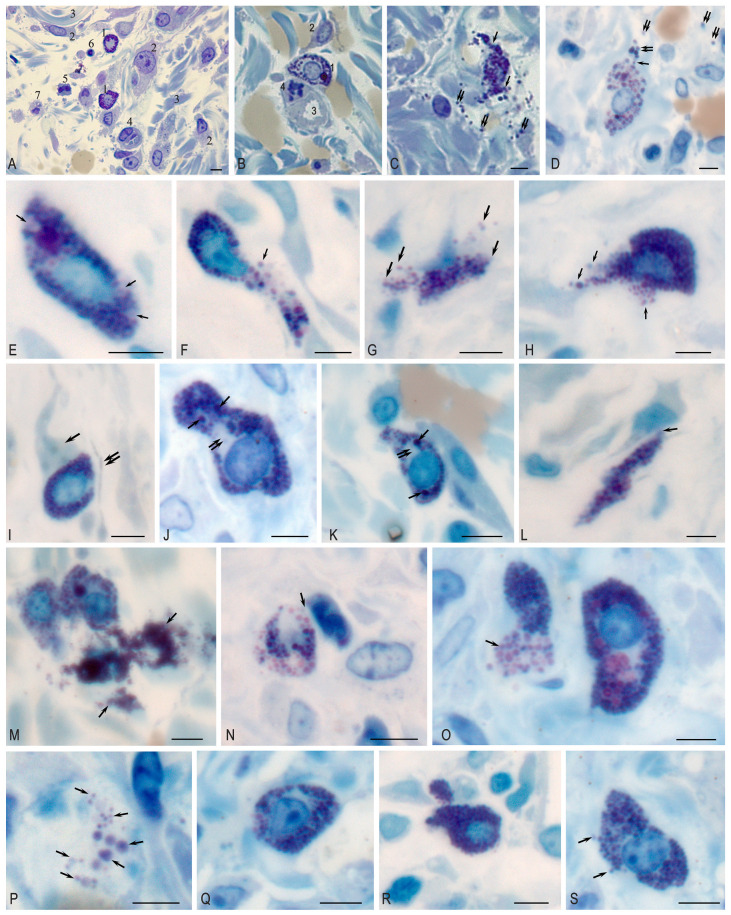
Mast cell in the purulent wound area. Technique: staining of semi-thin sections with Toluidine blue. (**A**–**C**) Day 1. (**A**) 1—non-degranulated mast cell; 2—fibroblast; 3—collagen fiber; 4—capillary; 5—leukocyte; 6—lymphocyte; 7—macrophage. (**B**) 1—non-degranulated mast cell; 2—fibroblast; 3—capillary lumen; 4—mitotic pericyte. (**C**,**D**) Exocytosis of mast cell granules (arrow); granules are located among collagen fibers (double arrow). (**E**–**H**) Day 3. (**E**) Mast cell with areas of cytoplasm free of granules (arrow). (**F**) The mast cell is filled with granules, with evidence of exocytosis of the granule contents into the extracellular matrix (arrow). (**F**–**H**) Different variants of whole-granular secretion (arrow). (**I**–**P**) Day 7. (**I**) Adjacent mast cell to fibroblast outgrowth (arrow). Telocyte cytoplasm is located nearby (presumably double arrow). (**J**,**K**) Heterogeneity in the sizes of secretory granules in the cytoplasm, some of which reach large sizes (arrow), with the formation of granule-free areas of cytoplasm (double arrow). (**L**) Adherence of mast cell to fibroblast (arrow). (**M**) Active secretory activity of mast cells with the formation of large extracellular conglomerates (arrow). (**N**) Colocalization of MC with neutrophil granulocyte (arrow). (**O**) Massive release of secretory granules from the MC (arrow). (**P**) Freely localized autonomous secretory granules of MC of different sizes in the extracellular matrix (arrow). (**Q**–**S**) Day 12. (**Q**) TC without signs of secretory granule release. (**R**) MC filled with secretory granules, with low degranulation activity. (**S**) Low activity of granule secretion into the extracellular matrix (arrow). Scale: 5 µm.

On observation day 1 (which is day 3 after the formation of the purulent wounds), there were signs of a complicated inflammatory process with purulent–fibrinous plaque, pronounced edema around the wound defect, necrosis in some parts with a tendency to spread beyond the simulated wound, with dilated capillaries filled with blood, and purulent–necrotic masses. MCs with large, bright metachromatic granules were noted in loose connective tissues between collagen fibers, white adipocytes, and vessels. MCs were located diffusely without the formation, of clusters, were identified by a pronounced polymorphism (Figure 1A), and were mainly in two functional states: non-granulated (in which granules densely filled the cytoplasm of cells) and in the state of degranulation (in which granules were located in the intercellular space) (Figure 1B,C). The degranulation index was 44.0% (Table 4). The number of MCs per unit area of the wound was 35.48 ± 5.66.

In addition, microscopy of semi-thin sections made it possible to identify some secretory and histotopographic patterns of MC in the local tissue microenvironment during wound regeneration. Various secretion patterns were detected on day 3, including the release of secretome components by the exocytotic mechanism (Figure 1E), as well as whole-granular secretion (Figure 1F–H). By day 7, the highest secretory activity was formed in the experimental group (Table 4), which was accompanied by histotopographic features. A close proximity with fibroblasts and telocytes (Figure 1I,L), granular leukocytes (Figure 1N) was observed. From the cytotopographic point of view, secretory granules in the same cell differed significantly in size and were selectively located in the cytoplasm (Figure 1J,K). Secretory activity was the highest compared to other observation periods (Figure 1M,O), which led to the accumulation of secretory granules in the extracellular matrix (Figure 1P). By day 12 of the experiment, MC degranulation decreased (Figure 1Q–S).

**Figure 2:**
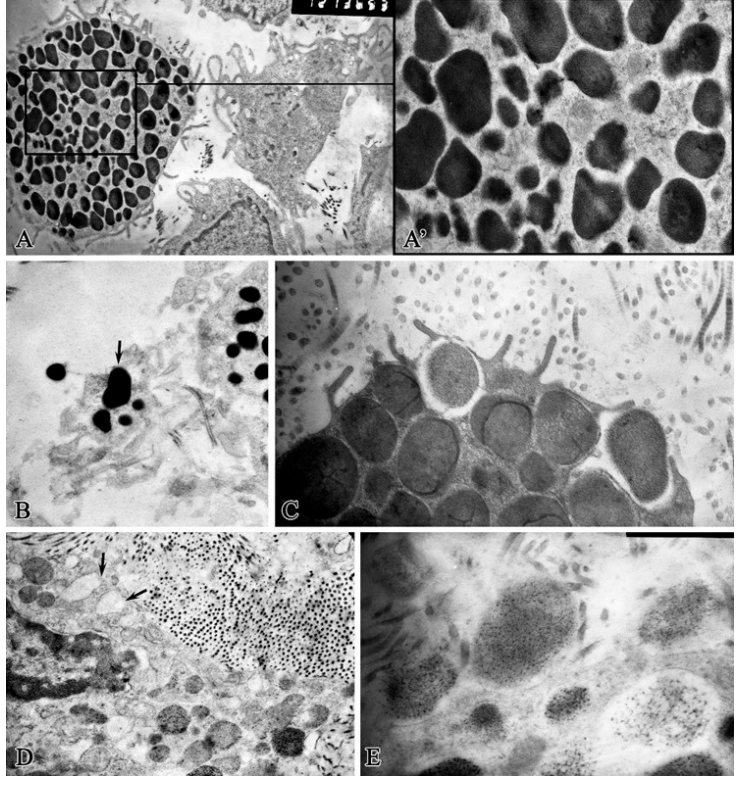
Structural and functional state of mast cells in the bottom of a purulent wound on observation day 1 before treatment. (**A**)—non-granulated mast cell; (**A′**)—fragment of Figure 2 (**A**)—granule polymorphism, granule fusion; (**B**)—cytoplasmic clasmatosis with MCs granules pointed to by an arrow; (**C**)—whole-granular degranulation stage; (**D**)—cavities formed due to degranulation (arrows); (**E**)—extracellular location of mast cell granules and among collagen fibrils. Magnification: (**A**)—12,000; (**A′**)—36,000; (**B**–**D**)—23,000; (**E**)—46,000.

Electron microscopic examination show that the cytoplasm of MCs in the non-granulated state was densely filled with polymorphic electron-dense granules, ranging in size from 0.1 to 1.2 microns^2^ (Figure 2A). In the central part of the cell, small granules without a pronounced membrane merged with each other, which is one of the stages of maturation of MC granules (Figure 2A′). Mature electron-dense granules with a denser “core” were observed along the cell periphery.

Cellular organelles are represented by single mitochondria and granular cytoplasmic reticulum profiles. Many thin cytoplasmic sprouts were detected on MC surfaces, with which intercellular interactions with endothelial cells, single fibroblasts, and macrophages, as well as cell–matrix interactions with collagen fibrils were facilitated.

Rapid whole-granular secretion was observed in two variants: as a result of clasmatosis, lacing of an autonomous fragment of the MC cytoplasm with granules and then granule secretion (Figure 2B); and granule secretion into the extracellular space, where they lost their electron density, swelled, and dissolved.

The second variant of whole-granular MC secretion involved the movement of the granule as close as possible to the cell periphery, then a break in the MC plasmalemma is observed, followed by the granule wholly exiting into the intercellular space (Figure 2C), while a narrow strip of MC cytoplasm covers the resulting cavity (Figure 2D). During mass degranulation, the MCs looked like shadows. In the extracellular space, the granules were located among the collagen fibrils and interacted with them (Figure 2E).

Microbial contamination of wounds decreased in the experimental group on observation day 3—in 2 subjects out of 5, it was below the critical level (10^5^), in the control group—only in 1 subject (Table 1). In the control group, the spread of necrosis and edema were noted. In the experimental group, as a result of effective cleaning of wounds from microbial and cellular detritus using a medication with 0.1% polyhexanide solution for treatment, the healing process was more active than in the control group. In the wound base, young granulation fibroblasts also appear, young proliferating fibroblasts along with functionally active fibroblasts synthesizing collagen were found, macrophages and mast cells were also noted in the granulation fibroblast, which indicated the replacement of leukocyte phase of regenerative inflammation by the macrophage phase. The number of MCs per unit area increased by 1.3 times, and then by almost 2 times compared to the control group in the previous observation period. MC degranulation index increased by 1.6 times compared to the previous period; when comparing MC degranulation index in the experimental group with the control group, it was slightly higher (Table 4).

**Figure 3:**
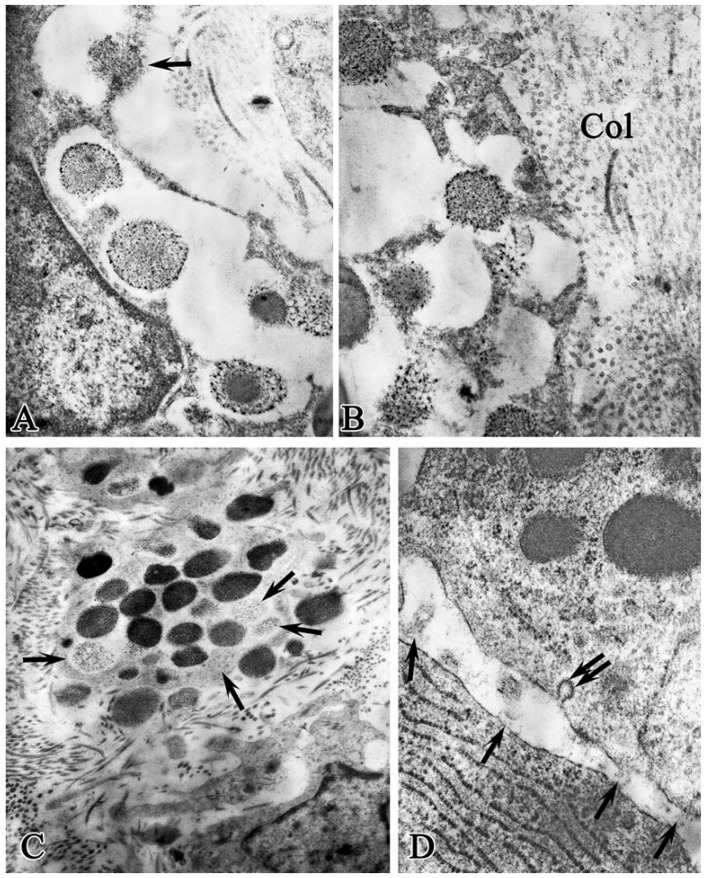
Structural and functional state of mast cells in the purulent wound base in the experimental group on observation day 3. (**A**)—Loss of perigranular membranes, release of granule contents into the intercellular space (arrow); (**B**)—loss of perigranular membranes, formation of labyrinths, partial dissolution of granules; Col—extracellular collagen; (**C**)—low electron density of mast cell granules (arrow); (**D**)—intercellular contact between MCs and fibroblast. Fine-grained material connecting two mast cells (single arrow); caveola (double arrow); magnification: (**A**,**B**)—28,000; (**C**)—14,000; (**D**)—46,000.

In the experimental group during= this observation period, the prevalence of non-rapid whole-granular degranulation of MCs was noted, as it was during the previous observation period, while the so-called gradual or “diffuse degranulation”, or “differential release”, was noted. The ultrastructural picture of this process is presented in the form of fusion of perigranular membranes and the formation of peculiar labyrinths in which granules of low electron density are located, up to barely distinguishable contours, i.e., the granule contents are dissolved in lacunae and are secreted into the intercellular matrix containing the partially dissolved granules (Figure 3A–C). In the control group, whole-grain degranulation prevailed, as in the previous study period.

The interaction of MCs with fibroblasts in the experimental group occurred by means of cellular surface proximity of the cells (simple contact) (Figure 3D). At the same time, in the narrow intercellular space, regions of electron-dense small-granular material were noted, which seemed to join the cells together. In addition, flask-shaped caveolae were found on the surface of the MC plasma membrane, which may be responsible for the transmission of cellular signals localized in caveoles (growth factors, receptors, and other molecules involved in intracellular signaling). Intercellular contacts also occurred through long cytoplasmic outgrowths of cells, and also, quite often, MC granules were located next to the plasma membrane of fibroblasts.

In the control group, similar ultrastructural patterns were less common.

On observation day 7, the level of microbial contamination of purulent wounds in four subjects in the experimental group was lower than critical, while in the control group, this was observed in one subject (as in the previous observation period). In the wounds of subjects in the experimental group, active maturation of granulation tissue was observed. Vertical loops of blood vessels were detected among horizontally oriented collagen-synthetizing fibroblasts and collagen fibrils. In this period, the maximum number of MCs per unit area and degranulated MCs, DI 80.1%, were noted (Table 4).

In the control group, wound defects were filled with immature granulation tissue with a small number of fibroblasts and newly formed vessels without pronounced orientation in comparison to the wound surface with moderate neutrophilic infiltration.

**Figure 4:**
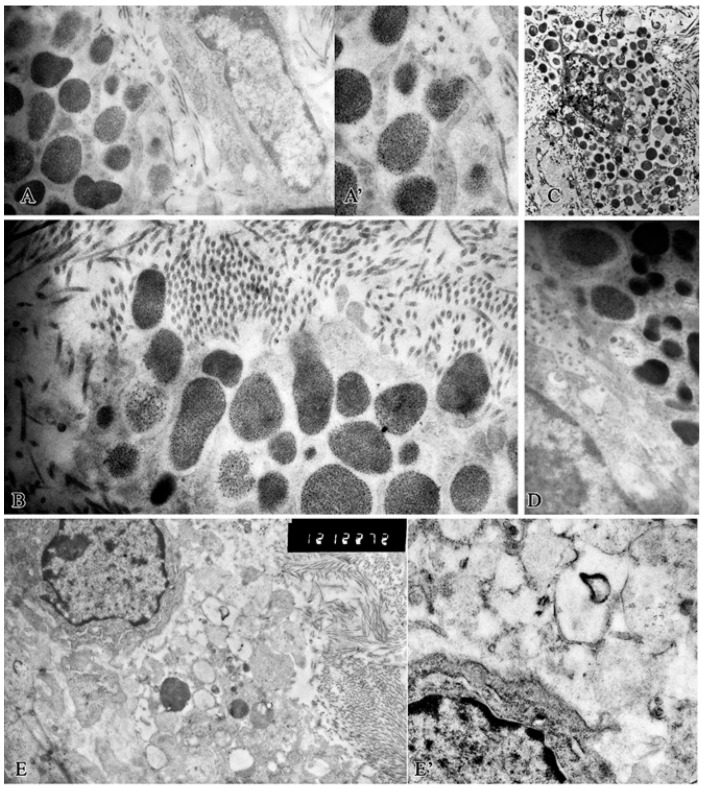
This The structural and functional state of mast cells in the purulent wound base on observation day 7 in the experimental group. (**A**)—A variant of gradual degranulation of MCs with the formation of a labyrinth, exocytosis of MCs granules into the intercellular matrix. (**A′**)—fragment of (**A**), exocytosis of granules and contact with collagen fibrils in a nearby fibroblast; (**B**)—variants of secretory mechanisms. (**C**)—black silver grains (^3^N-uridine) above the nucleus, indicating RNA synthesis in degranulating MCs; (**D**)—contact between MCs and fibroblast; (**E**)—degranulating MCs among collagen interact with fibroblast. The cytoplasm of MCs is filled with partially emptied granules; (**E′**)—is a fragment of Figure 4E. The MC plasmalemma is broken. The granule contents are in contact with the fibroblast surface. Magnification: (**A**)—17,000; (**A′**)—23,000; (**B**)—17,000; (**C**)—9000; (**D**)—28,000; (**E**)—12,000; (**E′**)—36,000.

In the ultrastructural study of MCs in the experimental group, whole-granular secretion was observed in single cells. In general, gradual degranulation was observed; in the formed mazes there were granules with a lit matrix that could merge with each other, followed by exocytosis of granules into the intercellular matrix (Figure 4A,A′). Several secretory mechanisms were observed in the MCs (Figure 4B): whole-granular secretion and exocytosis. During exocytosis, secretory granules moved to the MC plasmalemma in the porosome region (permanent lipoproteinous structures of the plasmalemma), with which perigranular membranes converge and merge during secretion.

The contents of the secretory granule are released through the formed pore. This process is possible due to an increase in intravesicular pressure. This method of isolation by exocytosis through the porosome is called “Kiss and Run”. Multiple partial secretion from the secretory granule is performed by this method.

The formation of protogranules was noted, which indicated the process of formation and accumulation of secretory products in degranulating MCs (Figure 4B).

During this period, the maximum number of degranulated cells was noted. Electron microscopic radioautography over the core of an actively degranulating MC revealed an accumulation of black silver grains (^3^N-uridine), which indicated the synthesis of RNA in MCs, i.e., it was viable (Figure 4C).

MCs often came in contact with fibroblasts, which were the predominant cells in the granulation tissue (Figure 4A). These were both contacts of cell surfaces (Figure 4D) and MC granules with the surface of fibroblasts (Figure 4E,E′).

On the 12th day of observation in the experimental group, the epithelial shaft “crawls” on the regenerating tissue. The level of microbial contamination is lower than critical in 100% of the subjects of the experimental group, and in 60% of the subjects in the control group (in 3 out of 5 rats). In the experimental group, maturation of the fibrous stroma and reorganization of connective tissue were noted in the area of the wound defect. The number of fibroblasts and blood vessels decreases, the fibrous component prevails. The DI and number of MCs are less than in the control group and at the previous observation period.

In the control group of animals, diffuse lymphocytic infiltration of maturing granulation tissue with a predominance of fibroblasts and a small number of vessels was noted. The number of cellular elements predominated over the number of fibrous ones.

**Figure 5:**
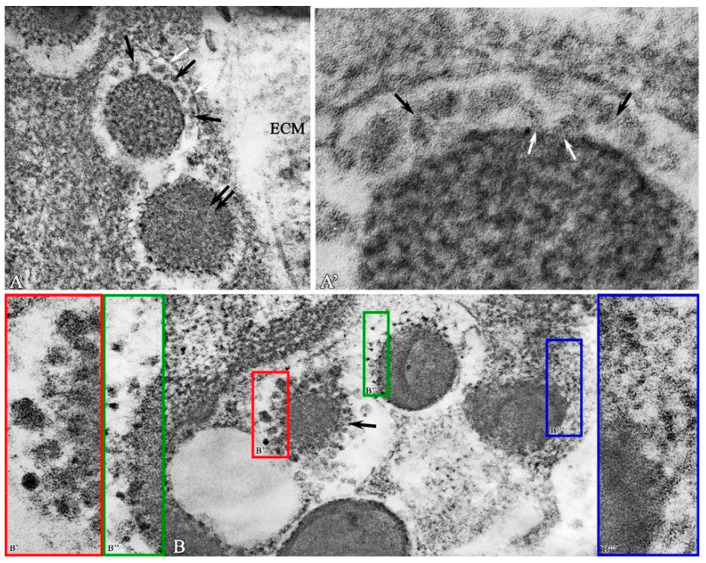
The structural and functional state of mast cells in the purulent wound base on observation day 12 in the experimental group. (**A**)—MC granules with partially preserved perigranular membrane (white arrow); fine-grained material between granules and perigranular membrane (black arrows), electron-dense granules (double arrow); extracellular matrix (ECM); (**A′**)—fragment of Figure 5A—fine-grained material between granules and perigranular membrane (black arrows) exit point of granular material from the secretory granule (white arrows); (**B**)—variants of ultrastructural organization of small granular material (black arrow) of secretory granules in the perigranular space: (**B′**)—association of small granular material into globules; (**B′′**)—small clusters in the perigranular space; (**B′′′**)—diffuse distribution of material in the perigranular space. Magnification: (**A**)—56,000; (**B**)—56,000; (**A′**,**B′**,**B′′**,**B′′′**)—220,000.

During electron microscopic examination of biopsies in the experimental group, ultrastructural signs of the gradual degranulation described above were noted. Features of the fine structure of secretory granules that were not detected at the previous observation periods were also found. Thus, in the narrow electron-transparent space between the perigranular membrane and the granular matrix, accumulations of fine-grained materials differing in electron density and size formed as a result of the decomposition of the dense matrix of a secretory granule were observed (Figure 5A,A′). A similar pattern was observed in the absence of a perigranular membrane (Figure 5B′,B′′,B′′′).

In the control group, MCs with signs of gradual degranulation prevailed over cells with rapid whole-granular degranulation.

## 3. Discussion

The presence of a high level of microbial contamination in purulent wounds inhibits reparative processes, and this is more often associated with biofilms in wounds [39,40], which protect microbial cells from external factors, including antibacterial drugs. This explains the severe course of the wound process and the continuing tendency to increase the number of long-term ongoing and recurrent processes [41]. Reparative regeneration in purulent wounds is characterized by a shift in the phases of the wound process over time, inhibition of collagenogenesis, slowing down of epithelialization of wounds, and a violation of the architectonics of fibrous tissue. Local treatment of infected wounds is based on several tasks: cleaning detritus, biofilms, and necrotic tissues, suppressing the infectious agent, and stimulating wound defect healing [42].

Various approaches are used to solve the above listed problems: the base or foundation of wound coating are developed, which ensures a dosed and prolonged release of the drug into the wound [43,44,45,46,47,48] and a search is also underway for the main active substance that is effective in combating biofilms.

The research is based on new approaches to identify and study biofilms, immune responses to biofilm-related infections, development of new antibiotics, changes in the tactics of antibiotic therapy, as well as the search for inhibitors of intercellular signaling, enzymes, and other methods of biofilm destruction [49,50,51,52,53].

For example, a combination of benzalkonium chloride and metronidazole with various bases, which include polyethylene oxide/sodium salt of carboxymethylcellulose/polymethylsiloxane polyhydrate, was shown to be effective [54]. Several other studies have suggested the use of pyolite-based disinfection therapy in combination with the artificial administration of necrophagous fly larvae [55,56,57].

**Figure 6:**
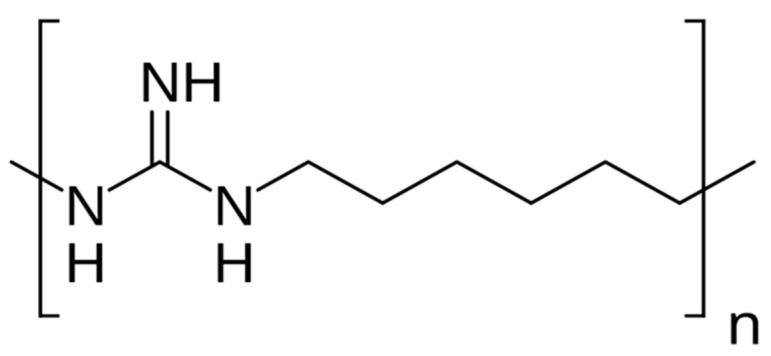
Chemical structure of polyhexamethylene guanidine [60].

The antimicrobial properties of biocidal compounds largely depend on their chemical structure and the structure of the microorganism’s cell, especially its cell wall and cytoplasmic membrane, which serve as an osmotic barrier regulating the selective penetration of substances into the cell. Polymers based on guanidine destroy the bacterial cell wall due to the electrostatic action of its positively charged molecules on the anionic groups of the bacterial cell wall [58]. This means that they have antimicrobial properties. In the treatment of superficial skin wounds in rodents, it was observed that the synthetic polymer polyhexamethylene guanidine hydrochloride (Figure 6) leads to a significant increase in the number of fibroblasts compared to the control group [59].

As our study showed, in the treatment of purulent wounds with a drug from the polyhexamethylene guanidine group with the antiseptic polyhexanide 0.1%, the level of microbial contamination significantly decreased on the 7th day of treatment and observation, and on the 12th day, bacteriological cultures were negative in all animals. In the control group of animals, a high degree of contamination (more than 10^5^ CFU/g) of purulent wounds was noted at all periods of observation. On day 12, in three of the five animals, the level of microbial contamination of wounds remained higher than the critical level (10^5^ CFU/g).

Decontamination of purulent wounds resulted in a decrease in signs of inflammation in the experimental group of animals. Stimulation of wound cleaning processes in the experimental group of animals was a necessary condition for the rapid transition of the wound process to the next phase, the proliferative phase, in which the granulation tissue develops and matures [61,62].

Studies have shown that bacteria in the biofilm are enclosed in a matrix of extracellular polymer substances synthesized by them, called quorum molecules; their phenotype is changed in comparison with single, planktonic cells [63,64]. Biofilm formation under wound defect conditions promotes the development of chronic inflammation and slows down healing [65,66]. In the study of Pundir P et al. (2019) evaluating the response of MCs to the competence-stimulating peptide (CSP)-1, a quorum molecule produced by Streptococcus pneumoniae, it was found that mast cells are able to detect CSP-1 via Mrgprb2, while MC activation occurs, which leads to growth suppression and antibacterial effects [67]. Thus, Mrgprb2 was found to be involved in MC-mediated bacterial cleansing in Streptococcus pyogenes and Staphylococcus aureus skin infections.

In addition, MCs are a fast and powerful inducer of inflammation. Activated MCs release various inflammatory mediators, such as histamine, tryptase, and vascular endothelial growth factor, which promote vasodilation, increase vascular permeability, and increase the migration of neutrophils, basophils, and monocytes outside the microcirculatory bed [9,13,68,69,70,71].

A study conducted on MC-deficient (MCD) mice compared to non-deficient mice showed a significant reduction in mobilized neutrophils in MCD mice [72].

Since MCs are a source of anti-inflammatory/immunosuppressive mediators (interleukin-10 (IL-10), transforming growth factor β (TGF-β), interleukins-10, -35) [73,74], it can be assumed that MCs may not only stimulate inflammation at the earliest stages of recovery, but also contribute to stopping the inflammatory response at later stages of healing [75,76,77,78]. Chemical mediators released by mast cells have also been shown to have a significant effect on the inflammatory healing phase [79,80,81].

Thus, the importance of MCs in bacterial cleansing and inflammatory response reinforces the importance of mast cells in the barrier function of the skin as an essential element of protection.

It is known that MCs are actively involved throughout wound healing [4,8]. The use of the drug with the antiseptic polyhexanide 0.1% leads to effective cleansing of purulent wounds. The size of the MC population and the degranulation index increased by the 3rd day of observation, which corresponded to the phase of inflammation of the wound process, and on the 7th day, in the proliferation phase, when the growth and maturation of MC granulation occurs. On day 12, during the remodeling phase of granulation tissue, these indicators decrease. Thus, in different phases of the wound process, degranulation of MCs and changes in their population size should be considered as an integral part of the compensatory-adaptive response to damage.

Considering that apart from mast cells, basophilic leukocytes could also enter the cohort of metachromatic cells when calculating the degranulation index, the nucleus structure (segmentation) and features of secretory granules (small number, large size) were used in order to objectively detect the basophilic leukocytes. Taken together, these characteristics of basophils made it possible to undoubtedly differentiate them from mast cells and not include them in the calculations.

As this study shows, rapid whole-granular degranulation prevailed in the first phase of the wound process-the inflammatory phase. When the mechanism of rapid degranulation is induced, the granules merge with each other and with the cell membrane, which provides an accelerated release of the contents. This type of degranulation is characteristic of acute processes and is mediated by the activation of the IgE–FceRI signaling pathway.

MCs play a crucial role in the second phase of the wound process—proliferation, when the formation and maturation of MC granulation occurs. This is the phase of active fibrillogenesis. As a result of ultrastructural rearrangement, fibroblasts actively produce intercellular matrix proteins, including collagen. Histamine directly increases fibroblast proliferation in vitro, while tryptase induces active movement of fibroblasts, mitotic cell division, and stimulates the synthesis of type I collagen by fibroblasts [9,33,82,83].

This explains the increase in the number of MCs in the growth and maturation phase of granulation tissue [84]. In our study, the number of MCs per unit area in the experimental group of animals was maximal on the 7th day of observation, which corresponded to the II phase of the wound process. The number of both direct and indirect MCs contacts with fibroblasts increases [4,85].

Alexandria Savage et al. [86] showed that MCs exosomes are an additional source of profibrous substances and represent a unique pathway for collagen generation. Thus, there are two parallel pathways in the MCs leading to fibroblast activation. One pathway is classical MC degranulation with the release of profibrotic mediators such as histamine and renin (ANG II) which act on resident fibroblast receptors that activate the collagen synthesis pathway [87,88,89]. Another pathway is the recently identified exosome pathway, in which MC exosomes are absorbed by the fibroblast cytosol, resulting in proline hydroxylation and an increased production of MCs and collagen [13,86].

In our study, the exosomal pathway for stimulating the collagen-synthetic activity of fibroblasts was not considered.

MCs are involved in such an important process as angiogenesis, which occurs in granulation tissue along with fibrillogenesis. VEGF, TGF-β, tryptase, and chymase, which are secreted by MCs, stimulate angiogenesis [90], while heparin, on the other hand, inhibits it [19,31,32,91,92].

Such subtle mechanisms of granulation tissue development are regulated by gradual MC degranulation, in which, as we have shown, selective release of mediators contained in granules occurs [93]. This type of secretion seems to be the main mechanism for the release of small doses of biologically active MCs substances during wound healing, angiogenesis, and fibrillogenesis (phase II of the wound process) [94,95].

Such diverse functions of MCs are performed as a result of constant cyclic changes in the cell, that is, the alternation of the processes of granule formation and maturation, their secretion and subsequent regranulation, which allows some authors to consider MCs as “single-celled glands” [96]. In our study, we noted the formation of protogranules, which indicated the process of formation and accumulation of secretory products in degranulating MCs.

In phase III of the wound process (day 12 of follow-up), the architectonics of collagen fibers are reorganized without a certain orientation to a parallel location relative to the wound surface. MCs participate in the formation of intercellular matter by synthesizing sulfated glycosaminoglycans (this elastic function of cells ensures the normal structure of connective tissue). As our study showed, the number of blood vessels and fibroblasts significantly decreased. The number of MCs and the degranulation index of MCs are significantly lower in the treatment of purulent wounds with an antiseptic drug compared to the control group, which confirms a decrease in the functional activity of MCs. In the untreated control, the number of MCs and the degranulation index continued to increase compared to the previous follow-up period.

Thus, the treatment of purulent wounds with an antiseptic drug contributed to a shift in the phases of the wound process while maintaining its general patterns; however, the activation of the process occurred at an earlier time than in the control group of animals without local treatment.

Inflammation, proliferation, and remodeling are the three main phases of wound healing that require complex cell–cell interactions. MCs are no longer considered “troublemakers” that cause allergies. MCs are universal cells capable of organizing and controlling several biological processes, interacting with microenvironmental cells. MCs are not only able to establish direct cell-to-cell contacts [75,76], but also interact via exosomes that carry mRNAs and microRNAs [97]. The functional consequences of exosome transfer and incorporation into recipient-cells (fibroblasts, endothelial cells) include enhancement, and possibly, acquisition of new functional properties [13,98].

Currently, it can be said that MCs are able to control the key events of wound healing: inflammation, proliferation, and remodeling of the extracellular matrix, which requires complex intercellular interactions (Table 5).

**Table 5:** Mast cell mediators involved in various stages of wound healing and their functions at each stage.

Phase	Mediators	Biological Effects
Hemostasis	Heparin	Inhibits the enzymatic and synthetic action of thrombin [99]. Binds molecules produced by mast cells [100].
	Tryptase	Suppresses thrombin-induced fibrinogen activity, which is responsible for blood clotting [25].
	TNF-α	Has a positive effect on the expression of coagulation factor XIIIa (fibrin stabilizing factor), so it promotes coagulation [84,101].
Inflammation	MCP-1s	Affects the phagocyte morphology [102].
Proliferation	Tryptase	Stimulates angiogenesis Cleaves fibronectin and activates PAR-2 [103,104].
	VEGF, TGF-β, Chymase	Stimulate angiogenesis [19,30,31,32,91].
	Heparin	Inhibits angiogenesis [30,32].
	Cytokines	Affects the phenotypic characteristics of activated fibroblasts [4].
Remodeling of the extracellular matrix	Tryptase	Promotes the synthesis of type I collagen [25,105].
	Histamine	Regulates skin remodeling processes [106,107]. Produces proteolytic enzymes [9,108].
	TGF-β1, TNF-α, IL-4	Regulates fibroblast proliferation [109].

## 4. Materials and Methods

### 4.1. Research Design

An in vivo experimental study was performed on 40 white male Wistar rats weighing 180.0 ± 20.0 g. All animals were kept under the same conditions in individual cages with free access to food and water. One of the standard variants of anesthesia (ketamine 5% 0.1–0.2 mg/kg per/m; relanium 0.5% 0.1–0.2 mg/kg per/m) was applied to the animals and the infected wound was modeled. A full-layer flap with a diameter of 16 mm was excised on the shaved back, fascia was dissected at the bottom of the wound, soft tissues were crushed once with a Kocher clamp, and a daily culture of St. aureus (AMCSC 25923) was introduced in the amount of 2.5 × 10^7^ CFU. The wound defect was closed with a sterile gauze cloth and fixed with a bandage.

The animals were divided into two groups, 20 in each (5 animals for each observation period in each group). In the first (control) group, no therapeutic agents were used, and a 2.0 cm × 2.0 cm gauze moistened with 0.9% sodium chloride solution was placed in the wound defect. In the second group, a gauze soaked in antiseptic (0.1% polyhexanide solution) was applied to the wounds. Biopsy samples (~1 mm^3^) were collected on day 3 after the formation of a purulent wound (observation day 1), then further collected on day 7 and 12 both in the experimental group and in the control group (i.e., the group without treatment).

### 4.2. Electron Microscopy

The collected biological materials were fixed in 2.5% glutaraldehyde solution and 1% osmium tetrachloride solution, dehydrated in alcohols of increasing concentration (50, 70, 96, and 100%), and then soaked in a propylene oxide–araldite resin mixture. Polymerization of araldite blocks took place in a thermostat at a temperature of 60 °C for two days. Metachromatic staining with toluidine blue (reaction of the interaction of a polyanionic heparin molecule with cationic dyes) of semi-fine sections with a thickness of 1.5–2 microns were used to identify MCs. Light microscopic examination of semi-thin sections was performed using a Leica DM 1000 microscope (Leica Microsystems, Wetzlar, Germany). The most significant parts of the materials were photographed using a Leica ICC50E camera. Ultrathin sections with a thickness of 100–200 nm were obtained using an ultramicrotome manufactured by LKB V (LKB, Stockholm, Sweden). Ultrastructural study of the preparations was performed using a JEM-2100 and JEM 100-CX electron microscope (JEOL, Tokyo, Japan) in transmission mode at an accelerating voltage of 80 KV. Images from the JEM 100-CX microscope were captured on film, and the negatives were analyzed after digitization using an Epson Perfection V850 Pro scanner (Epson, Nagoya, Japan).

A low-molecular-weight RNA precursor, ^3^H-uridine, was used in this study. To conduct an electron-radioautographic study, tissue pieces were incubated for 1.5 h in medium 199 containing 100 mCi/mL of ^3^H-uridine (specific radioactivity 26 Ci/mM). After pouring and cutting, semi-thin sections were covered with an “M” type photo emulsion and exposed for 3 days. After development with the D-19 developer, light-microscopic radio autographs were analyzed, then ultrathin sections were cut, which were covered with the same photo emulsion and exposed for 30–40 days at a temperature of 4 °C. After development and contrast, the sections were viewed under an electron microscope in transmission mode.

### 4.3. Quantitative Analysis

The area of wound defects was determined by digital planimetry using the ImageJ program ver. 1.54g.

The level of contamination of experimental wounds was interpreted by a semi-quantitative method with the definition of a high degree of contamination of wounds as more than 10^5^ CFU/g. For each drug, the number of MCs were counted in 20 viewpoints at magnification ×1000 (eyepiece ×10, lens ×100) with the area of one viewpoint being 0.031 mm^2^, followed by recalculation of the MC content in 1 mm^2^ of the biopsy sample, which was taken as the unit. MCs was identified by characteristic morphological features: oval shape, large size, small light oval nucleus, and metachromatic coloration. Granules in the MCs were mainly colored blue, purple (Figure 1), and blue with a reddish tinge.

The functional activity of MCs (degree of degranulation) was evaluated by the degranulation index (DI, %), which was calculated using Equation (1):

(1) $$DI=\frac{D}{D+N}\times 100$$

where *D* is the number of MCs with obvious signs of degranulation, *N*—the number of inactive MCs.

Comparisons of the average amount of MCs per unit area and the MC degranulation index were performed in relation to the control group at the same time of wound healing, as well as in relation to the corresponding indicator in sections of the previous period.

The healing rate was calculated with Equation (2):

(2) $$\frac{\left(S_{3}-S_{12}\right)\times 100\%}{S_{3}\times T}$$

S_3_—wound area on the 3rd day; S_12_—wound area on the 12th day; T—number of days between measurements (in this case, 9).

### 4.4. Statistical Analysis

Statistical processing of the obtained data was carried out using “Microsoft Office Excel 2003”. The data were presented as M ± m, where M is the sample mean and m is the average error. To determine the significance of the differences between the groups, the paired Student’s *t*-test was used. The difference between the groups was considered significant with an error probability of *p* < 0.05. Previously, all parameters in the study groups were checked for the normality of the distribution.

### 4.5. Ethical Review

All manipulations were performed in compliance with the requirements of the “European Convention for the Protection of Vertebrates Used for Experimental and Other Scientific Purposes” (Strasbourg, 1986), the declaration of the World Medical Association on the humane treatment of animals (Helsinki, 2000) and the recommendations and requirements of the Declaration of Helsinki and the approval of the Commission for Bioethical Control of the Keeping and Use of Laboratory Animals for Scientific Purposes of National Medical Research Radiological Center of the Ministry of Health of the Russian Federation (protocol No. 1-N-00034 issued on 14 April 2023).

## 5. Conclusions

The use of a drug from the group of polyhexamethylene guanidines with the antiseptic polyhexanide 0.1% for the treatment of purulent wounds quickly stops the inflammatory reaction and creates conditions for the development of the reparative abilities of granulation cells, primarily mast cells.

Structural and functional changes in the MC during the healing process correspond to the phase of the wound process and determine its course. In the inflammatory phase, rapid whole-granular degranulation of the MC with the secretion of biologically active proinflammatory substances that have a stimulating effect on inflammatory cells. Effective cleaning of purulent wounds is a necessary condition for the rapid transition of the wound process to the next phase—the proliferative phase. In the proliferation phase, the maximum number of MCs per unit area of wound tissue and the maximum degranulation index were noted. In the remodeling phase of granulation tissue, the number and functional activity of MCs sharply decrease, which contributes to the completion of the healing process by forming a fully fledged normotrophic scar. Gradual degranulation of MCs was characteristic in the phase of proliferation and remodeling.

Treatment of purulent wounds with a drug from the polyhexamethylene guanidine group with the antiseptic polyhexanide 0.1% contributed to a temporary shift in the phases of the wound process while maintaining its general patterns, while the activation of the process occurred at an earlier time than in the control group of animals without local treatment.

## Figures and Tables

**Table 1:** Number (in %) of animals with a high degree of microbial contamination of purulent wounds (more than 10^5^ CFU/g).

Observation Day	Control	Experimental
1	100	100
3	80	40
7	80	20 *
12	60	0 *

Note: * statistically significant intergroup values at *p* < 0.05 when compared with the control group.

**Table 2:** Phases of purulent wound healing in the experiment (day) (M ± m).

Phase of Wound Healing	Control	Experimental
Cleaning	9.6 ± 0.2	5.3 ± 0.3 *
Onset of granulation	11.7 ± 0.2	7.0 ± 0.3 *

Note: * statistically significant intergroup values at *p* < 0.05 in comparison with the control group.

**Table 3:** Change in the area of experimental wounds (mm^2^) (M ± m).

Observation Day	Control	Experimental
1	421.5 ± 0.9	421.2 ± 0.9
3	390.6 ± 0.9	370.5 ± 2.3
7	300.2 ± 2.3	241.8 ± 2.2 *
12	250.8 ± 4.2	150.8 ± 3.6 *

Note: * statistically significant intergroup values at *p* < 0.05 in comparison with the control group.

**Table 4:** Number of mast cells per unit area (M ± m) and degranulation index (%) in purulent wounds during the reparative regeneration process.

Observation Day	Control		Experimental	
	Number of MCs, pc	MC Degranulation Index, %	Number of MCs, pc	MC Degranulation Index, %
1	35.48 ± 5.66	44.0	35.48 ± 5.66	44.0
3	48.39 ± 8.71	63.2	64.52 ± 9.35	70.6
7	54.84 ± 7.74	66.7	80.65 ± 122.25 * ”	80.1
12	77.40 ± 7.74	71.8	48.39 ± 9.35 * ”	57.1

Note: * statistically significant intergroup values at *p* < 0.05 in comparison with the control group; ”—during comparison of the indicators with the previous observation period (*p* < 0.05).

## Data Availability

All data generated or analyzed during this study are included in this published article. Any additional inquiries may be directed to the corresponding author.

## Abbreviations

The following abbreviations are used in this manuscript:

MC	Mast Cell
CFU	Colony-Forming Unit
Ci	Curie
